# Supplementary material for: MetaRibo-Seq measures translation in microbiomes
Source: Nat Commun. 2020 Jun 29;11:3268. doi: 10.1038/s41467-020-17081-z (PMC7324362; doi:10.1038/s41467-020-17081-z)
Supplement: Supplementary file 10 — Supplementary Data 7 [file 41467_2020_17081_MOESM10_ESM.zip › File2/Confidence_High_Taxonomy/118029_out.krona.html]

Javascript must be enabled to view this page.

members
magnitude
magnitudeUnassigned
count
unassigned
taxon
rank

118029\_out

4

2
4
superkingdom

976
phylum
4

4
class
200643

order
4
171549

family
1
2005519

397864
1
genus

2033407

SRS147557\_contig\_number\_31894
1
species

2005525
3
family

375288
3
genus

1947048

SRS097958\_contig\_number\_12226
species
1

2
species

SRS045004\_contig\_number\_27794SRS045713\_contig\_number\_12032
46503
